# Correction: Huang et al. Correlation Study Between Dietary Behaviors, Lifestyle, and Psychological Problems in Chinese Children Aged 3–7. *Nutrients* 2025, *17*, 176

**DOI:** 10.3390/nu18121903

**Published:** 2026-06-12

**Authors:** Zixuan Huang, Jiamin Han, Ying Jiang, Shiming Li, Gang Wang, Zhenhe Zhou, Haohao Zhu

**Affiliations:** 1 Affiliated Mental Health Center of Jiangnan University, Wuxi 214151, China; 7242808008@stu.jiangnan.edu.cn (Z.H.); 6242828002@stu.jiangnan.edu.cn (J.H.); jiangying1010@jiangnan.edu.cn (Y.J.); mhclsm@163.com (S.L.); zhuhh@jiangnan.edu.cn (H.Z.); 2 State Key Laboratory of Food Science and Technology, Jiangnan University, Wuxi 214122, China; wanggang@jiangnan.edu.cn

In the original publication [1], there was a mistake in the Informed Consent Statement. The original Informed Consent Statement was intended to refer to the waiver of signing consent for publication, not to imply that informed consent for participation was not obtained. Informed consent was obtained from all legal guardians of the pediatric participants (ages 3–7). The corrected Informed Consent Statement appears below:

**Informed Consent Statement:** Informed consent was obtained from all legal guardians. Consent for publication was waived as it was included in the original informed consent form, which stated that results could be published without additional consent if no sensitive personal information was involved.

The authors state that the scientific conclusions are unaffected. This correction was approved by the Academic Editor. The original publication has also been updated.
